# Widening access for graduates: Policies, pathways and technology

**DOI:** 10.1111/medu.70069

**Published:** 2025-10-14

**Authors:** Kirsty Alexander, Dylan Smith, Joshua Cleaver, Anton Zhyzhyn

**Affiliations:** ^1^ School of Medicine University of Dundee Dundee UK; ^2^ School of Medicine University of St Andrews St Andrews UK; ^3^ ‘You Can Be a Doctor’ Charity Edinburgh UK

## Abstract

Reflecting on the complexity of pursing medicine as under‐represented minorities, the authors point to ways in which graduate programs can be expanded by widening policy definitions and targets.

Acknowledging the complexities of widening access (WA) to medicine, Bartle et al.'s^1^ realist review unpicks what outcomes a diverse range of WA interventions are aiming for and how these are operationalised. The review reveals that most interventions focus on shaping underrepresented minority (URM) applicants' dispositions (e.g. attitudes and motivations) and/or adjusting their situations (e.g. their individual circumstances), rather than looking inward to institutional factors, including the mismatch between the potential applicant's needs and the WA opportunities presented by the institution. The authors argue that, without a move to acknowledge and address institutional factors, progress in WA will continue to be slow paced and limited. In essence, it is not possible to achieve equity and inclusion by expecting only one side to change. This prompted us to think closely about institutional factors that limit WA to medicine and changes that could be made to meet URM applicants' needs.

URM graduates pursuing medicine as a second degree often encounter complex and overlooked challenges. In numerous countries, medical education is primarily designed for applicants to enter as school leavers, but individuals with university‐level qualifications may also apply. In the United Kingdom, however, graduate applicants are not typically flagged as a URM applicant in the same way school leavers are and are ineligible for most medical schools' WA interventions.^4^

URM graduates pursuing medicine as a second degree often encounter complex and overlooked challenges.

This is a clear mismatch between the institutional offering and URM applicant need: It is naïve to suggest that the factors that influence a URM applicant's chances to enter, and successfully complete, medical education and training will be dramatically changed by obtaining a degree. Although a degree may provide an URM applicant with the requisite academic requirements, the systemic and social factors that influenced their choices earlier in life persist into, and through, medical school.^2^, ^3^ Moreover, graduates are often carrying substantial debt from their first degree and have more independent living commitments. So what can be done to support URM graduate applicants?

First, this issue needs to be addressed at the root cause. Governmental policy provides a catalyst for change by setting desired outcomes and narrowing the avenues available to achieve these. UK governmental WA policies focus their outcomes on applicants under the age of 21. Medical schools follow suit, focusing their efforts on avenues to widen access to school leavers. Stakeholders should push policymakers to widen their policy definitions and targets so that URM graduate applicants are included. They should work with policymakers to find workable solutions for the logistical challenges of applying comparable WA metrics to applicants at different life stages. Unlike URM indicators preferred in other countries (e.g. ethnicity, indigenous or aboriginal background), the United Kingdom's dominant focus on postcode‐determined socioeconomic status is not so easily translated between life stages. Moreover, the literature has long argued that using this measure alone is too narrow to be truly meaningful.^4^

Stakeholders should push policymakers to widen their policy definitions and targets so that URM graduate applicants are included.

Second, we encourage the continued and expanded provision of graduate entry medicine (GEM) programmes or pathways (also known as ‘fast track’ or ‘accelerated’ programmes). These aim to be holistically tailored to support the academic skills and social needs of graduates and are increasingly being developed in countries where the standard entry is still directly from school (e.g. the United Kingdom, Australia, New Zealand, Poland and South Africa). Studies show GEM students perform equally to those on standard courses.^5^ Initial indications suggest that GEM programmes may have a greater proportion of URM students^6^, ^7^ although larger, multi‐site studies are required.

We encourage the continued and expanded provision of graduate entry medicine (GEM) programmes or pathways.

One significant attraction to URM applicants may be that GEM programmes are shorter, thus lowering financial cost. In the United Kingdom, as elsewhere, there is substantially less governmental financial support for a second undergraduate degree. URM individuals who may have felt that medicine was unsustainable as a first degree due to the cost are even less likely to consider completing medicine as a graduate. For this reason, institutional and governmental funding systems should be revised to support GEM students (see, for example, the ScotGEM fee reductions and bursary^8^). The impact of these on enabling URM graduates into, and through medical education, should be carefully evaluated.

Third, across all countries and contexts, URM applicants need to be made more aware of the various paths into medicine. Bartle et al.'s^1^ review highlights that WA interventions rely on potential applicants coming forward for activities, rather than striving to make these attractive and attainable to those who have never previously considered medicine. This problem is exacerbated for graduates in countries where entry is predominantly designed for, and advertised to, school leavers. Institutional change is required to raise awareness of the graduate pathways, particularly within undergraduate programmes that have greater numbers of URM students. Basic information should become common knowledge, for example, that degree classification is used instead of school grades for academic entry requirements and that a science degree is not necessarily required.

Institutional change is required to raise awareness of the graduate pathways.

In an increasingly interconnected world, there is a potential to democratize knowledge by moving content online. Digital resources may be more visible, and potential applicants only require a small degree of motivation and/or curiosity to access content in a low‐cost, low‐risk way that can be juggled around other commitments. We require a greater understanding about the online sources URM applicants use to learn about pathways into medicine, how influential each is and why each is valued. This also requires institutional change: Bartle et al.^1^ highlight that only a minority of interventions involved community collaboration. Institutions should acknowledge that including and acting on these viewpoints is essential to enable accessibility.

In an increasingly interconnected world, there is a potential to democratize knowledge by moving content online.

We do know, however, that URM applicants desire peer‐to‐peer support^9^ and that they are increasingly accustomed to accessing content directly from its creator rather than through an institutional filter. WA interventions could thus consider focusing more attention on informing influencers on commonly used forums (e.g. Reddit, Discord, TikTok). There may be a reluctance for the institutional change required to pass over some control to these less familiar, less regulated and more chaotic forms of communication,^10^ especially due to concerns over misinformation. However, we are seeing some universities experimenting, for example, with informal interviews on their TikTok accounts. Lessons from the Covid‐19 years suggest that online interactions cannot fully replace the in‐person experience of an WA intervention.^11^ Nonetheless, if co‐designed content reaches and positively influences URM individuals who would otherwise not have considered medicine, that is a first step. The next is to clear the pathway through policy and system change.

## AUTHOR CONTRIBUTIONS

**Kirsty Alexander:** Conceptualization; writing—original draft; writing—review and editing. **Dylan Smith:** Conceptualization; writing—review and editing. **Joshua Cleaver:** Conceptualization; writing—review and editing. **Anton Zhyzhyn:** Conceptualization; writing—review and editing.

## CONFLICT OF INTEREST STATEMENT

The authors have no conflicts of interest to declare.

## Data Availability

Data sharing is not applicable to this article as no datasets were generated or analysed during the current study.

## References

[1] Bartle, E , Carr, SE , Olson, R , et al. Widening access to medicine: A realist review. Med Educ. 2025; 1‐13. doi:10.1111/medu.70017 PMC12805216 40778559

[2] Ashley L , McDonald I . When the penny drops: understanding how social class influences speciality careers in the UK medical profession. Soc Sci Med. 2024;348:116747. doi:10.1016/j.socscimed.2024.116747 38547804

[3] Krstić C , Krstić L , Tulloch A , Agius S , Warren A , Doody GA . The experience of widening participation students in undergraduate medical education in the UK: a qualitative systematic review. Med Teach. 2021;43(9):1‐1053. doi:10.1080/0142159X.2021.1908976 33861176

[4] The MSC Selection Alliance . Good practice in contextual admissions. The Medical Schools Council. 2018. https://www.medschools.ac.uk/wp-content/uploads/2025/05/good-practice-in-contextual-admissions.pdf

[5] Shehmar M , Haldane T , Price‐Forbes A , et al. Comparing the performance of graduate‐entry and school‐leaver medical students. Med Educ. 2010;44(7):699‐705. doi:10.1111/j.1365-2923.2010.03685.x 20636589

[6] James D , Ferguson E , Powis D , Symonds I , Yates J . Graduate entry to medicine: widening academic and socio‐demographic access. Med Educ. 2008;42(3):294‐300. doi:10.1111/j.1365-2923.2008.03006.x 18275417

[7] Garrud P , McManus IC . Impact of accelerated, graduate‐entry medicine courses: a comparison of profile, success, and specialty destination between graduate entrants to accelerated or standard medicine courses in UK. BMC Med Educ. 2018;18(1):250. doi:10.1186/s12909-018-1355-3 30400933 PMC6219209

[8] University of St Andrews . Scottish Graduate Entry Medicine (ScotGEM) MBChB. 2025. https://www.st-andrews.ac.uk/subjects/medicine/scotgem-mbchb/

[9] Sartania N , Alldridge L , Ray C . Barriers to access, transition and progression of Widening Participation students in UK Medical Schools: The students' perspective. MedEdPublish. 2021;10(1):132. doi:10.15694/mep.2021.000132.1 38486567 PMC10939539

[10] Rainford J . Moving widening participation outreach online: challenge or opportunity? Perspectives: Policy and Practice in Higher Education. 2021;25(1):2‐6. doi:10.1080/13603108.2020.1785968

[11] Canovan, C. & Alcock, J. (2002). More students. More engagements. Less impact? Assessing the effectiveness of online versus in‐person widening participation delivery. University of Central Lancashire. https://lancashirefutureu.org.uk/wp-content/uploads/2023/02/More-students-more-engagement-less-impact-FINAL.pdf

